# Case Report: Acquired Disseminated BCG in the Context of a Delayed Immune Reconstitution After Hematological Malignancy

**DOI:** 10.3389/fimmu.2021.696268

**Published:** 2021-08-03

**Authors:** Vincent Gies, Yannick Dieudonné, Florence Morel, Wladimir Sougakoff, Raphaël Carapito, Aurélie Martin, Noëlle Weingertner, Léa Jacquel, Fabrice Hubele, Cornelia Kuhnert, Sophie Jung, Frederic Schramm, Pierre Boyer, Yves Hansmann, François Danion, Anne-Sophie Korganow, Aurélien Guffroy

**Affiliations:** ^1^Department of Clinical Immunology and Internal Medicine, National Reference Center for Systemic Autoimmune Diseases (CNR RESO), Tertiary Center for Primary Immunodeficiency, Strasbourg University Hospital, Strasbourg, France; ^2^Université de Strasbourg, INSERM UMR-S1109, Institut thématique interdisciplinaire (ITI) de Médecine de Précision de Strasbourg, Transplantex NG, Faculté de médecine, Fédération Hospitalo-Universitaire OMICARE, Fédération de Médecine Translationnelle de Strasbourg (FMTS), Strasbourg, France; ^3^Université de Strasbourg, Faculty of Pharmacy, Illkirch, France; ^4^APHP.Sorbonne Université, Hôpital Pitié-Salpêtrière, Laboratoire de Bactériologie-Hygiène, Centre National de Référence des Mycobactéries et de la Résistance des Mycobactéries aux Antituberculeux (CNR-MyRMA), Paris, France; ^5^Sorbonne Universités, Inserm, Centre d'Immunologie et des Maladies Infectieuses (Cimi-Paris), UMR 1135, Paris, France; ^6^Immunology Laboratory, Strasbourg University Hospital, Strasbourg, France; ^7^Department of Infectiology, Strasbourg University Hospital, Strasbourg, France; ^8^Departement of Pathology, Strasbourg University Hospital, Strasbourg, France; ^9^Departement of Nuclear Medicine and Molecular Imaging, ICANS, University Hospital of Strasbourg, Strasbourg, France; ^10^Department of Internal Medicine, Strasbourg University Hospital, Strasbourg, France; ^11^Hôpitaux Universitaires de Strasbourg, Centre de Référence Maladies Rares Orales et Dentaires (O-Rares), Pôle de Médecine et de Chirurgie Bucco-Dentaires, Strasbourg, France; ^12^Laboratory of Bacteriology, Strasbourg University Hospital, Virulence bactérienne Précoce UR7290-Lyme Borreliosis Group, FMTS-CHRU Strasbourg, Institut de Bactériologie, Strasbourg, France

**Keywords:** BCG, BCGosis-susceptible PIDs, Immunodeficiency, hematological malignancies, contamination, case report

## Abstract

**Context:**

Disseminated infections due to *Mycobacterium bovis* Bacillus Calmette-Guérin (BCG) are unusual and occur mostly in patients with inborn error of immunity (IEI) or acquired immunodeficiency. However, cases of secondary BCGosis due to intravesical BCG instillation have been described. Herein, we present a case of severe BCGosis occurring in an unusual situation.

**Case Description:**

We report one case of severe disseminated BCG disease occurring after hematological malignancy in a 48-year-old man without BCG instillation and previously vaccinated in infancy with no complication. Laboratory investigations demonstrated that he was not affected by any known or candidate gene of IEI or intrinsic cellular defect involving IFNγ pathway. Whole genome sequencing of the BCG strain showed that it was most closely related to the *M. bovis* BCG Tice strain, suggesting an unexpected relationship between the secondary immunodeficiency of the patient and the acquired BCG infection.

**Conclusion:**

This case highlights the fact that, in addition to the IEI, physicians, as well as microbiologists and pharmacists should be aware of possible acquired disseminated BCG disease in secondary immunocompromised patients treated in centers that administrate BCG for bladder cancers.

## Introduction

Disseminated infections due to *Mycobacterium bovis* Bacillus Calmette-Guérin (BCG) are rare and occur in three types of conditions. First, it occurs in rare cases of inborn errors of immunity (IEI) involving phagocytosis, such as chronic granulomatous diseases (CGD), or interferon-gamma (IFNγ) pathways (called Mendelian susceptibility to mycobacterial diseases or MSMD) at the time of BCG vaccination. The second situation concerns cases associated with acquired immunodeficiency to IFNγ pathways that are described as a phenocopy of IEI. They lead to opportunistic infections (fungi, parasites, and bacteria), tuberculosis, and infection due to non-tuberculosis mycobacteria (1–4). Finally, reactivations are described in immunocompromised patients with human immunodeficiency virus (HIV) (5, 6), and cases of secondary disseminated BCG disease (BCGosis) are also reported under BCG therapy for bladder malignancy (7, 8). Here, we described a case of severe secondary BCGosis occurring after hematological malignancy in an adult without BCG instillation and previously vaccinated in infancy with no complication.

## Case Description

A 48-year-old Caucasian man was referred to our department for progressive asthenia, anorexia, and cachexia (−12 kg in 9 months).

**Figure 1:**
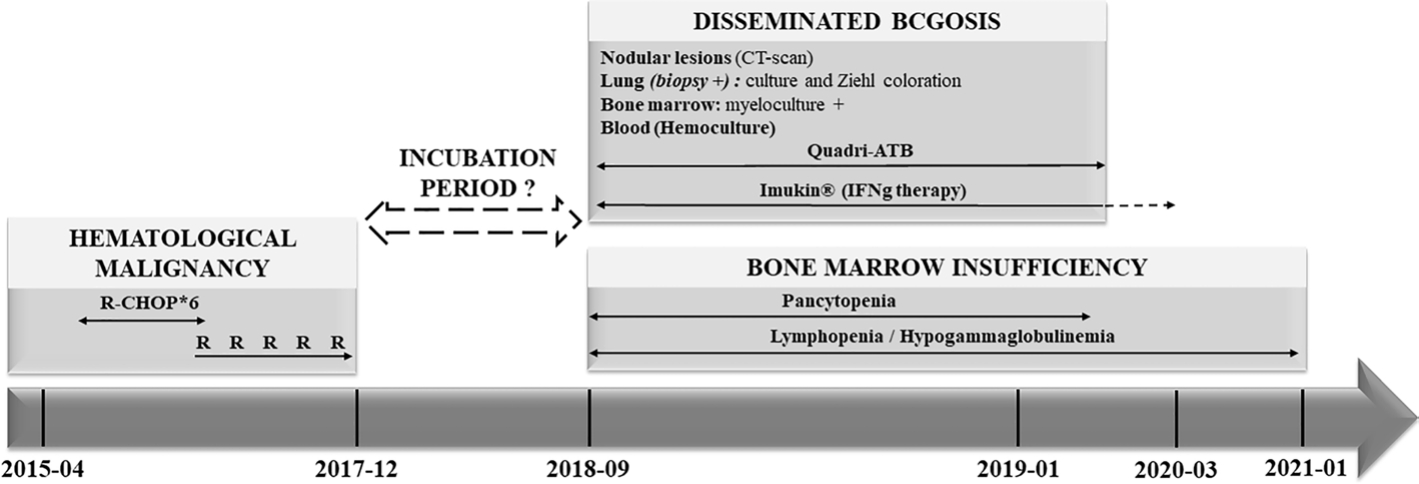
Clinical history (Timeline). R, Rituximab; CHOP: C, cyclophosphamide; H, hydroxyadriamycine; O, oncovin^®^; P, prednisone; CT, computerized tomography; ATB, antibiotherapy.

His main medical history was a hematological malignancy (OMS 2008 grade 1–2 follicular lymphoma) diagnosed 3 years before and involving the lacrimal gland and the bowel. Biopsies (right part of the colon) revealed a monotypic lymphoid infiltrate made of CD20+, CD5−, CD10+, and Bcl6+ lymphocytes with a Ki67 index at 50%. Lungs were not involved at this time (**Supplementary Figure S1**). He was treated with combined chemotherapy (R-CHOP) and a maintenance therapy with Rituximab every 2 months, for 18 months. He had experienced neither opportunistic and severe infections nor autoimmune diseases. Timeline of his medical history is resumed in **Figure 1**.

**Figure 2:**
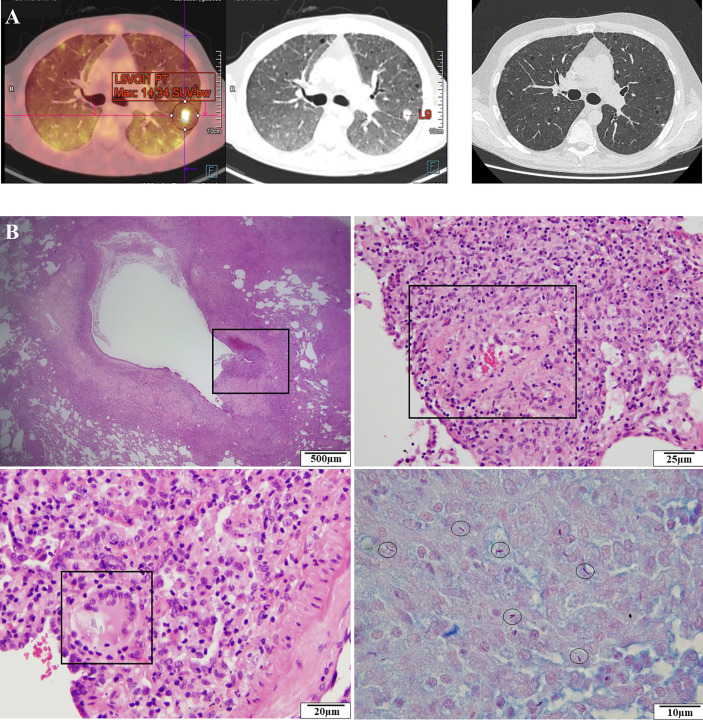
Clinical evolution and histological analysis of the patient. **(A)** 18F-FDG-TEP-CT-scan revealing an interstitial lung disease with nodular lesions (left). CT-scan normalization after 3 months (right). **(B)** Histological analysis showing a peri-bronchial granulomatous inflammation with ulceration (upper left), a perivascular granuloma (upper right), giant multinucleated cells (lower left), and BAAR with Ziehl-Neelsen staining (lower right).

Seven months after the last Rituximab therapy, the patient presented fever, splenomegaly (17 cm), and fine dry crackles at pulmonary auscultation with dyspnea (New York Heart Association Classification class II–IV). An interstitial lung disease with nodular lesions was diagnosed on CT-scan with a decline of alveolar exchanges on breath tests (**Figure 2A**). Broncho-alveolar lavage (BAL) revealed an alveolar lymphocytosis (230,000 cells/ml, 67% lymphocytes, 28% macrophages) with inverted CD4/CD8 ratio and without any virus (screened by PCR), bacteria, or parasite in culture. Biology showed inflammatory syndrome, acute renal failure, pancytopenia with severe lymphopenia, mild neutropenia, thrombopenia and anemia, as well as hypogammaglobulinemia (**Supplementary Table S1**). Bone marrow aspiration revealed a poor cellularity and fat involution (reflecting likely the nutritional deficiency state) without malignant cells. Considering kidney failure, a biopsy was made and revealed an acute interstitial nephritis without glomerular lesions. 18F*-*FDG PET*/*CT-scan showed several hypermetabolic lesions (tonsil, lung nodules, and parenchyma) but no hypermetabolism of the spleen. Within a couple of days, the condition of the patient worsened with hypoxemia requiring increasing level of oxygen despite probabilistic antibiotherapy (sulfamethoxazole/trimethoprim for *Pneumocysis jirovecii* PCR+ in BAL and large-spectrum antibacterial therapy with piperacillin/tazobactam). Corticosteroids were added and transiently improved the respiratory state. A lung biopsy was performed that finally revealed invasive infection by *M. bovis* BCG, suspected by Ziehl-Neelsen coloration and confirmed by PCR and culture (**Figure 2B**). The *M. bovis* BCG strain was also found in the bone marrow and blood cultures. The strain was phenotypically susceptible to rifampicin, isoniazid, and ethambutol. *Cryptococcus neoformans* was also identified in lung biopsy but without central nervous system involvement. HIV PCR and serology were negative. Phagocytosis assays were normal, discarding the hypothesis of chronic granulomatous disease.

Despite a treatment regimen of isoniazid, rifampin, pyrazinamide, and ethambutol for BCGosis, initiated before the strain identification, and fluconazole for *Cryptococcus*, the patient state worsened with need for intensive care unit support. Corticosteroids were re-introduced, and G-CSF therapy was added two times per week. We hypothesized a primary or secondary defect in IFNγ pathway, such as an autoimmunization against IFNγ, and we decided to introduce Imukin^®^ as an add-on therapy (50 µg S.C. three times/week and after 2 weeks at 100 µg S.C. three times/week) (**Supplementary Table S1**). This strategy finally allowed an improvement of the patient’s condition with CT-scan and TEP-scan complete normalization after 3 and 6 months, respectively (**Figure 2A**). Interferon therapy was maintained during 12 months with bi-antibiotherapy (rifampcin and isoniazid) and fluconazole after the complete remission in order to avoid any relapse, and 12 months after treatment’s discontinuation, the patient remained in complete remission.

### Functional Analysis

The patient harbored a severe post-chemotherapy lymphopenia with delayed immune reconstitution of B-cell and T-cell compartment, i.e., very low level of CD19^+^ cells with a selective lack of IgM production, and low levels of naive CD45RA+ T cells. CD4/CD8 ratio was conserved in proportion (**Supplementary Table S1**). The absolute number of monocytes was normal with 55% of classical, 18% of intermediate, and 27% non-classical monocytes.

**Figure 3:**
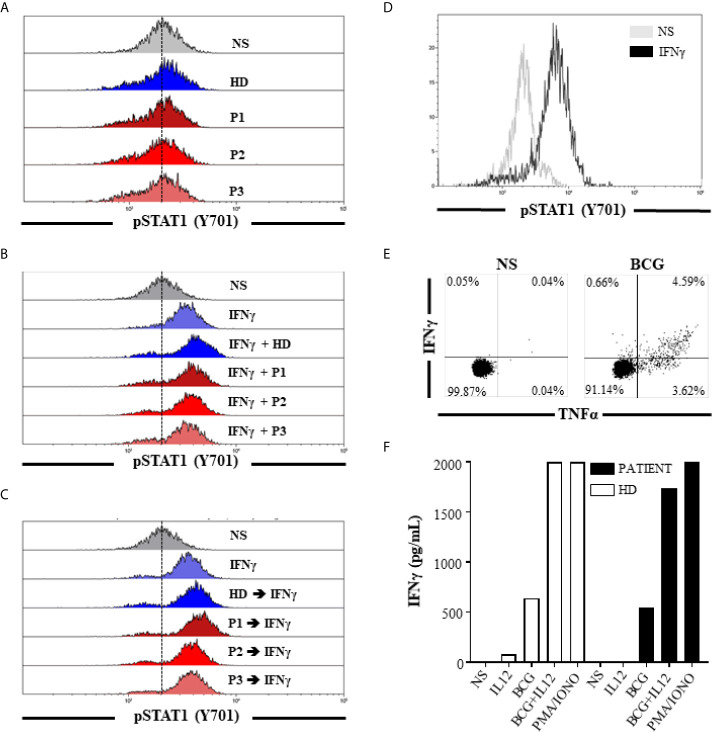
Absence of IFNγ or other autoantibodies that may directly hinder/block IFNγ signaling and normal response to IFNγ or BCG stimulation. STAT1 phosphorylation (Y701) of HD monocytes after stimulation for 15 min **(A)** with 25% (v/v) allogenic HD or patient’s serum (from different time points: P1, P2, and P3) and **(B)** with IFNγ previously mixed with 25% (v/v) allogenic HD or patient’s serum (from different time points: P1, P2, and P3). **(C)** STAT1 phosphorylation (Y701) of HD monocytes after IFNγ stimulation for 15 min. Cells were preincubated 30 min at room temperature in the presence of 25% (v/v) HD or patient’s serum (from different time points: P1, P2, and P3) and washed before IFNγ stimulation. **(D)** STAT1 phosphorylation (Y701) of monocytes from the patient after IFNγ stimulation for 15 min. **(E)** Frequency of IFNγ^+^ and/or TNFα^+^ CD4^+^ T cells from the patient after no stimulation or stimulation with heat inactivated BCG for 48 h. **(F)** IFNγ concentration in culture supernatant after no stimulation, IL12, heat-inactivated BCG, or PMA/IONO stimulation of PBMC from the patient and one HD for 48 h. BCG: bacillus Calmette-Guérin; HD, healthy donor; IFNγ, interferon gamma; NS, non-stimulated; P1, patient’s serum before BCGosis diagnosis and IFNγ therapy (2018-08); P2, patient’s serum at the time of BCGosis diagnosis and during IFNγ therapy (2018-11); P3, patient’s serum after BCGosis diagnosis and IFNγ therapy (2019-02).

Potential IFN pathway defects that may lead to ineffective response towards BCG were explored. IFNγ stimulation of peripheral mononuclear cells (PBMCs) from a healthy donor (HD) in the presence of 25% (v/v) allogenic HD or patient’s serum (from different time points, i.e., before, during, and after IFNγ therapy) did not impair STAT1 phosphorylation. Similarly, no significant difference in STAT1 phosphorylation was observed when we mixed the IFNγ and the patient’s serum before using it to stimulate PBMCs from HD. These results suggested for the absence of anti-IFNγ autoantibodies or any other autoantibodies that may directly hinder/block IFNγ signaling (**Figures 3A–C**). Absence of anti-GM-CSF autoantibodies was also confirmed (data not shown). STAT1 phosphorylation, after IFNγ stimulation of monocytes from the patient, was preserved (**Figure 3D**). CD4^+^ T cells from the patient responded to BCG (**Figure 3E**) and showed no apparent defect of IFNγ production after BCG ± IL12 stimulation (**Figure 3F**). Altogether, these results did not support the hypothesis of an IEI or acquired immunodeficiency related to defective host defense mechanisms against BCG.

### Genomic Analysis

Whole exome sequencing (WES) was performed but did not reveal/detect any known mutations or unknown variants with high *in silico* predicted consequence (CADD > 10, MAF < 0.005%) involved in MSMD (i.e., *IL12RB1, IL12B, IL12RB2, IL23R, IFNGR1, IFNGR2, STAT1, CYBB, IRF8, SPPL2A, TYK2, ISG15, RORC, JAK1*, and *NEMO*) (9). Similarly, no candidate genes of IEI were found (**Supplementary Table S2**).

**Figure 4:**
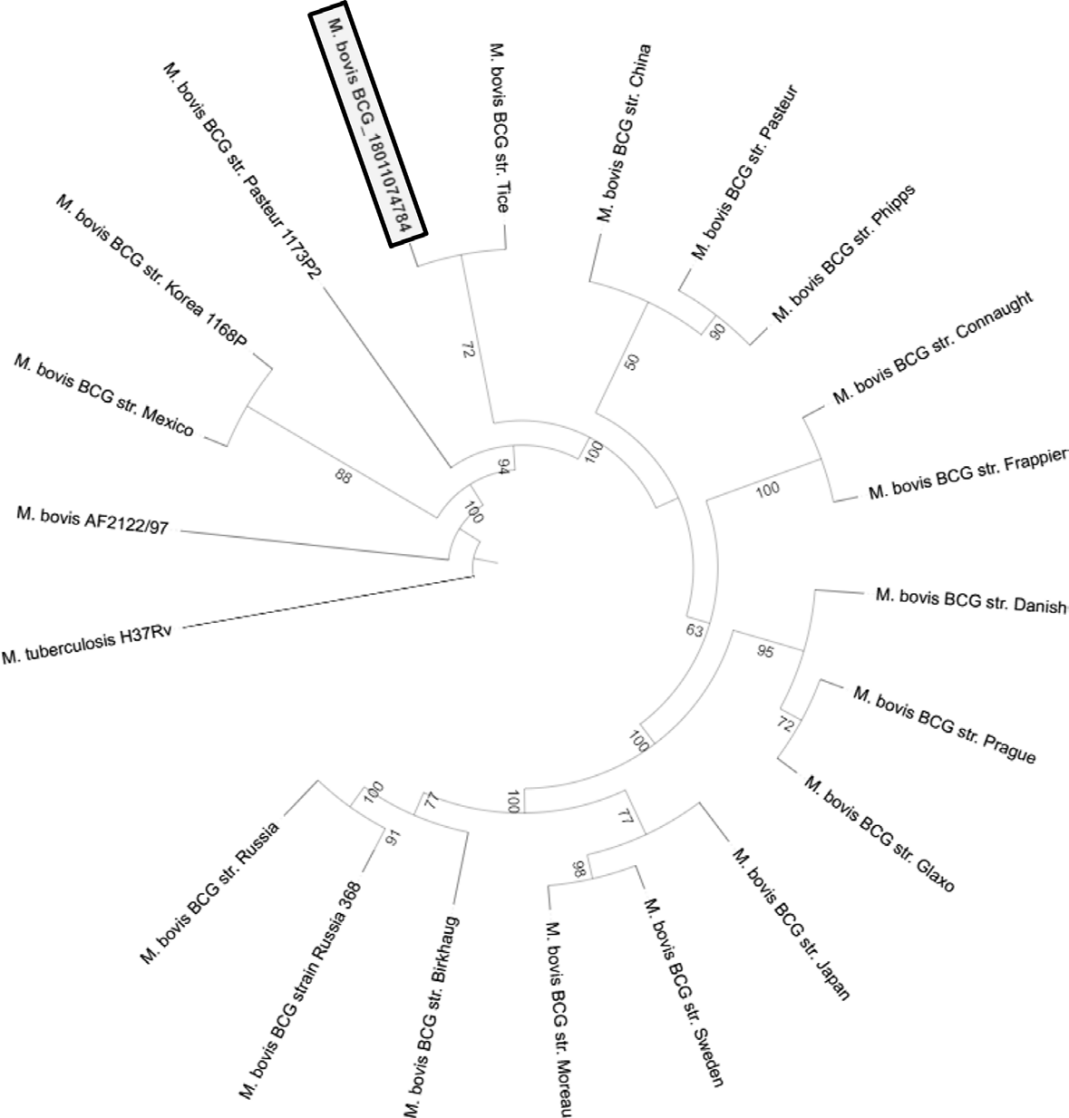
Maximum likelihood phylogenetic tree representing the relationship between the clinical *M. bovis* BCG strain (*M. bovis* BCG_1811074784) and reference *M. bovis* BCG strains. Bootstrap values of each branch are indicated.

Whole genome sequencing (WGS) of *M. bovis* BCG strain and comparative phylogenomic analysis with the genomes of reference *M. bovis* BCG strains were performed at the National Reference Center for Mycobacteria and Resistance of Mycobacteria to Antituberculosis (CNR-MyRMA). The results showed that the patient’s strain (*M. bovis* BCG_ 1811074784, **Figure 4**) was most closely related to the *M. bovis* BCG Tice strain. 

## Discussion

Because of the opportunistic features of infection (severe BCGosis, *Cryptococcus*) and considering the remission of the patient after IFNγ therapy introduction, we suspected a late-onset IEI. However, no rare monogenic causes of genetic susceptibility to tuberculosis, or genetic risk factor such as P1104A *TYK2* allele (10), were found; neither did we find other known pathogenic variants involved in IEI. Although we cannot totally exclude the existence of some unknown genetic susceptibility factors not evidenced by our WES analysis, the fact that the patient was vaccinated several times (because of anergy to the intradermoreaction test) with BCG vaccine during infancy and did not develop any symptom argues against an IEI. Moreover, none of his relatives were infected by BCG or other mycobacteria, even after exposition to BCG for vaccination. Additionally, no intrinsic defect in IFNγ production or IFNγ response was detected. Anti-cytokine autoantibodies are known to be a cause of adult-onset infection susceptibility (also called “phenocopies” of IEI) (1, 11–13), but the presence of anti-IFNγ or anti-GM-CSF has been excluded.

Finally, WGS and comparative phylogenomic analysis of the isolated *M. bovis* BCG revealed that this clinical strain was more closely related to the *M. bovis* BCG Tice. *M. bovis* BCG vaccine strains were derived by the repeated *in vitro* passage of *M. bovis* leading to its attenuation. The original BCG strain was distributed to many laboratories all around the world, which, in turn, carried out subcultures, generating daughter strains named according to their geographical origin. The BCG Tice strain is actually used as adjunctive therapy for superficial bladder cancer (OncoTICE^®^). Intravesical instillation of BCG for bladder cancer has been previously reported to be responsible for disseminated BCGosis in both immunocompromised and immunocompetent patients (7, 8, 14–16). The delay for BCGosis development is usually lower than 1 year, and some authors argued that the strain used in bladder installation could correlate with the frequency and severity of BCGosis (17). However, no vesical instillations were performed in our case and the results of the phylogenomic comparison might suggest the possibility of an acquired BCG infection during one of the chemotherapy sessions with an infection that remained latent during several months before becoming invasive. Interestingly, some cases of BCGosis have also been reported in patients with hematological malignancies who had never received intravesical BCG instillation (18–21), and a possible role of iatrogenic infection through central catheter was suggested (22) (**Supplementary Table S3**). Most of these cases were already linked to BCG Tice strain; the possible mechanism could be environmental contamination through aerosol generation during intra-vesical instillation or colonization of the outside of the central catheter by colonized gloves (18–21).

## Perspectives

We report herein one case of severe BCGosis, possibly linked to intrahospital acquisition of the BCG Tice strain. We excluded the possibility of an underlying IEI and we showed an unexpected relationship between the secondary immunodeficiency of the patient, due to the lymphoma and its treatment, and the acquired BCG infection. Using IFNγ therapy in association with antibiotherapy, corticosteroids, and G-CSF, the patient progressively recovered with a complete remission after several months. Although only assumptions about the origin of this contamination can be made from this single case, physicians, especially hematologists and oncologists, as well as pharmacists and microbiologists should be aware of the risk of disseminated BCGosis in immunocompromised patients treated in centers that administrate BCG for bladder cancers.

## Data Availability Statement

The original contributions presented in the study are included in the article/**Supplementary Material**. Further inquiries can be directed to the corresponding author.

## Ethics Statement

The patient’s written consent was obtained.

## Author Contributions

VG, A-SK, and AG contributed to conception and design of the study. YD, FM, WS, AM, NW, LJ, FH, CK, SJ, FS, PB, YH, FD, A-SK, and AG contributed to the collection of the data. VG, YD, FM, WS, RC, FS, PB, and AG performed the analysis. VG and AG wrote the first draft of the manuscript. YD, FM, WS, RC, AM, NW, LJ, FH, CK, SJ, FS, PB, YH, FD, A-SK, and AG wrote sections of the manuscript. All authors contributed to the article and approved the submitted version.

## Conflict of Interest

The authors declare that the research was conducted in the absence of any commercial or financial relationships that could be construed as a potential conflict of interest.

## Publisher’s Note

All claims expressed in this article are solely those of the authors and do not necessarily represent those of their affiliated organizations, or those of the publisher, the editors and the reviewers. Any product that may be evaluated in this article, or claim that may be made by its manufacturer, is not guaranteed or endorsed by the publisher.
