# Supplementary material for: Case Report: Acquired Disseminated BCG in the Context of a Delayed Immune Reconstitution After Hematological Malignancy
Source: Front Immunol. 2021 Aug 3;12:696268. doi: 10.3389/fimmu.2021.696268 (PMC8369751; doi:10.3389/fimmu.2021.696268)
Supplement: Supplementary file 1 [file DataSheet_1.pdf]

CASE REPORT: ACQUIRED DISSEMINATED BCG IN THE CONTEXT OF A DELAYED IMMUNE RECONSTITUTION AFTER HEMATOLOGICAL MALIGNANCY

SUPPORTING INFORMATION

AUTHORS & AFFILIATION

Vincent Gies[1,2,3], Yannick Dieudonné[1,2], Florence Morel[4,5], Wladimir Sougakoff[4,5], Raphaël Carapito[2,6], Aurélie Martin[7], Noëlle Weingertner[8], Léa Jacquel,[1,2] Fabrice Hubele[9], Cornelia Kuhnert[10], Sophie Jung[2,11], Frederic Schramm[12], Pierre Boyer[12], Yves Hansmann[7], François Danion[7], Anne-Sophie Korganow[1,2], Aurélien Guffroy[1,2]*.

[1]Department of Clinical Immunology and Internal Medicine, National Reference Center for Systemic Autoimmune Diseases (CNR RESO), Tertiary Center for Primary Immunodeficiency, Strasbourg University Hospital, F-67000 Strasbourg, France.

[2]Université de Strasbourg, INSERM UMR - S1109, Institut thématique interdisciplinaire (ITI) de Médecine de Précision de Strasbourg, Transplantex NG, Faculté de médecine, Fédération Hospitalo-Universitaire OMICARE, Fédération de Médecine Translationnelle de Strasbourg (FMTS), F-67000 Strasbourg, France.

[3]Université de Strasbourg, Faculty of Pharmacy, F-67400 Illkirch, France.

[4]APHP.Sorbonne Université, Hôpital Pitié-Salpêtrière, Laboratoire de Bactériologie-Hygiène, Centre National de Référence des Mycobactéries et de la Résistance des Mycobactéries aux Antituberculeux (CNR-MyRMA)

[5]Sorbonne Universités, Inserm, Centre d'Immunologie et des Maladies Infectieuses (Cimi-Paris), UMR 1135, Paris, France

[6]Immunology Laboratory, Strasbourg University Hospital, F-67000 Strasbourg, France

[7]Department of Infectiology, Strasbourg University Hospital, F-67000 Strasbourg, France.

[8]Departement of Pathology, Strasbourg University Hospital, F-67000 Strasbourg, France.

[9]Departement of Nuclear Medicine and Molecular Imaging, ICANS, University Hospital of Strasbourg, F-67000 Strasbourg

[10]Department of Internal Medicine, Strasbourg University Hospital, F-67000 Strasbourg, France

[11]Hôpitaux Universitaires de Strasbourg, Centre de Référence Maladies Rares Orales et Dentaires (O-Rares), Pôle de Médecine et de Chirurgie Bucco-Dentaires, 67000 Strasbourg, France

[12]Laboratory of Bacteriology, Strasbourg University Hospital. Virulence bactérienne Précoce UR7290-Lyme borreliosis group, FMTS-CHRU Strasbourg, Institut de Bactériologie, F-67000 Strasbourg

*corresponding author

CORRESPONDANCE:

Dr. Aurélien Guffroy, MD, PhD

Department of Clinical Immunology and Internal Medicine

National Reference Center for Autoimmune Diseases,

Hôpitaux Universitaires de Strasbourg

Strasbourg, France.

Telephone number: 0033 369555021

Fax: 0033 369551835

E-mail address: aurelien.guffroy@chru-strasbourg.fr

MATERIAL & METHODS

Patients and healthy donors

The study was conducted in accordance with the principles of Helsinki declaration. Written informed consents were obtained from the patient as well as from age and gender matched healthy donors (HD).

Phagocytosis assays

The investigation of phagocytosis and of oxidative burst activity were performed at the hospital laboratory (CE/IVD methods PHAGOTEST™, PHAGOBURST™, Celonic) according to the manufacturer's protocols.

PBMC stimulation and cytokine detection

Heparinized blood samples were collected from HD. Peripheral blood mononuclear cells (PBMC) were separated by density gradient centrifugation. Cells were plated at 10^6 cells/well in a 24 well-plate containing RPMI supplemented with 10% Fetal Bovine Serum (FBS) and stimulated for 48 hours with phorbol myristate acetate (PMA; 100ng/mL; Invivogen) and ionomycin (1µg/mL; Invivogen), IL12 (100ng/mL; Preprotech), or heat-inactivated patient's strain of *Mycobacterium Bovis* bacillus Calmette-Guérin (1 CFU/cell). Culture supernatants were collected from the stimulated PBMCs after 48 hours, and cryopreserved. Later, thawed supernatant was used to measure IFNγ levels by ELISA following the manufacturer's instructions (Invitrogen)

<u>Intracellular cytokines detection:</u> Protein transport inhibitor cocktail (eBioscience) was added during the last 6 hours of incubation. Surface staining was then performed during 15 minutes at +4°C. Fixation and permeabilization were performed with BD Cytofix/Cytoperm (BD) according to the manufacturer's recommendations. Samples were incubated with anti-cytokines

antibodies for 30 minutes at +4°C. Cell viability was assessed by initial incubation with Fixable Viability Dye eFluor 780 (eBioscience) following the manufacturer's protocol.

Cells were acquired on a Gallios flow cytometer (Beckman Coulter) and data analysis was performed using Kaluza software (Beckman Coulter) The following monoclonal anti-human antibodies, purchased from BD Biosciences, were used: CD3 (UCHT1), CD4 (RPA-T4), CD8 (RPA-T8), CD14 (MϕP9), IFNγ (B27), TNFα (MAb11).

Phospho-STAT1 (pSTAT1) assessment

Fresh EDTA peripheral blood samples were immediately subjected to density gradient centrifugation to get mononuclear cells suspensions. PBMCs were starved during 16 hours in serum free medium (at 37°C). $0.5*10^6$ PBMC were then stimulated for 15 minutes with IFNγ (1000UI/mL (50 ng/mL); Preprotech) or 25% (v/v) HD, patient serum (from different time points). If indicated (i) PBMCs were preincubated 30 minutes at room temperature in the presence of 25% (v/v) HD or patient serum (from different time points) and washed before IFNγ stimulation, or (ii) PBMC were stimulated with a solution containing IFNγ preincubated 30 minutes at room temperature with 25% (v/v) HD or patient serum (from time points). Cells were then labeled for pSTAT-1(Y720) and surface markers using BD Phosflow™ Fix Buffer I and BD Phosflow™ Perm Buffer III (BD Biosciences) following the manufacturer's recommendations. The cells were acquired on a Gallios flow cytometer (Beckman Coulter) and data analysis was performed using Kaluza software (Beckman Coulter) The following monoclonal anti-human antibodies, purchased from BD Biosciences, were used: CD3 (UCHT1), CD4 (RPA-T4), CD14 (MϕP9), STAT1-pY701 (4a).

Whole Genome Sequencing (WGS) of *M. bovis* BCG strain and phylogenomic analysis

The *M. bovis* BCG strain was sent to the French National Center for Mycobacteria (NRC-MyRMA) for expertise. Total genomic DNA was extracted using the geneLEAD VIII (Diagenode Diagnostics®) automated DNA extractor from isolate grown on Löwenstein-Jensen medium. Preparation of paired-end libraries using an Illumina Nextera XT kit (Illumina, San Diego, CA, USA) and Illumina NextSeq 550 WGS sequencing were done by the sequencing platform P2M-PIBnet (Institut Pasteur, Paris, France) following manufacturer's recommendations. The raw data of Illumina sequencing reads have been deposited in the National Center for Biotechnology Information (NCBI) Sequence Read Archive (SRA) database (SRA accession number SRR13767788, BioProject ID PRJNA704236).

The WGS raw data were analyzed with BioNumerics 7.6® (Applied Maths) and on the Galaxy server. The quality of paired end reads was checked with FastQC (version 0.11.7)

The genomes of the vaccinal *M. bovis* BCG strains, *M. bovis* reference genome AF2122/97 were downloaded from PATRIC (https://www.patricbrc.org/) for genomic comparison and phylogenetic analysis. Alignment of protein sequences was performed with MUSCLE and CDS nucleotide sequences (1000 genes) with BioPython (Codon_align function). The Bootstrapping analysis was realized with RAxML (x100). The phylogenetic tree was generated according to the Maximum Likelihood method and visualized and annotated on iTOL (Interactive Tree of life) (https://itol.embl.de).

Whole Exome Sequencing

As previously described (1), genomic DNA was isolated from patient's and parents' whole peripheral blood using standard protocols. Exome sequencing libraries were prepared with the Twist Library preparation kit and captured with Human Core Exome probes extended by Twist Human RefSeq Panel (Twist Bioscience, San Francisco, CA) following the manufacturer's recommendations. Paired-end (2 × 75 bp) sequencing was performed on a NextSeq500

sequencer (Illumina, San Diego, CA). Before any processing, quality control was performed using FastQC. The raw reads data were next mapped using the Burrows-Wheeler Alignment (BWA) tool. Average target read coverage was at least 60-fold. After read mapping, further quality indicators were calculated from the resulting BAM file using SAMtools, Qualimap. Variant calling was done using the GATK HalotypeCaller of the GATK software suite. The annotation was performed by VEP, the Ensembl Variant Effect Predictor. We focused only on protein-altering variants (missense, nonsense, splice site variants and coding indels) with alternative allele frequencies < 0.005 in the 1000 Genomes Project, the Genome Aggregation Database (gnomAD) and an internal exome database including ~1000 exomes.

BIBLIOGRAPHY

1. Guffroy A, Solis M, Gies V, Dieudonne Y, Kuhnert C, Lenormand C, Kremer L, Molitor A, Carapito R, Hansmann Y, et al. Progressive multifocal leukoencephalopathy and sarcoidosis under interleukin 7: The price of healing. *Neurol Neuroimmunol Neuroinflamm* (2020) **7**: doi:10.1212/NXI.0000000000000862

2. Meije Y, Martínez-Montauti J, Caylà JA, Loureiro J, Ortega L, Clemente M, Sanz X, Ricart M, Santomà MJ, Coll P, et al. Healthcare-Associated Mycobacterium bovis-Bacille Calmette-Guérin (BCG) Infection in Cancer Patients Without Prior BCG Instillation. *Clin Infect Dis* (2017) **65**:1136–1143. doi:10.1093/cid/cix496

3. Coppes MJ, Olivieri NF, Howes M, Pusic M, Gold R, Richardson SE. Mycobacterial brain abscess possibly due to bacille Calmette-Guérin in an immunocompromised child. *Clin Infect Dis* (1992) **14**:662–665. doi:10.1093/clinids/14.3.662

4. Stone MM, Vannier AM, Storch SK, Peterson C, Nitta AT, Zhang Y. Meningitis Due to Iatrogenic Bcg Infection in Two Immunocompromised Children. *New England Journal of Medicine* (1995) **333**:561–563. doi:10.1056/NEJM199508313330905

5. Waecker NJ, Stefanova R, Cave MD, Davis CE, Dankner WM. Nosocomial transmission of Mycobacterium bovis bacille Calmette-Guerin to children receiving cancer therapy and to their health care providers. *Clin Infect Dis* (2000) **30**:356–362. doi:10.1086/313652

6. Vos MC, de Haas PEW, Verbrugh HA, Renders NHM, Hartwig NG, de Man P, Kolk AHJ, van Deutekom H, Yntema JL, Vulto AG, et al. Nosocomial Mycobacterium bovis-bacille Calmette-Guérin infections due to contamination of chemotherapeutics: case finding and route of transmission. *J Infect Dis* (2003) **188**:1332–1335. doi:10.1086/379034

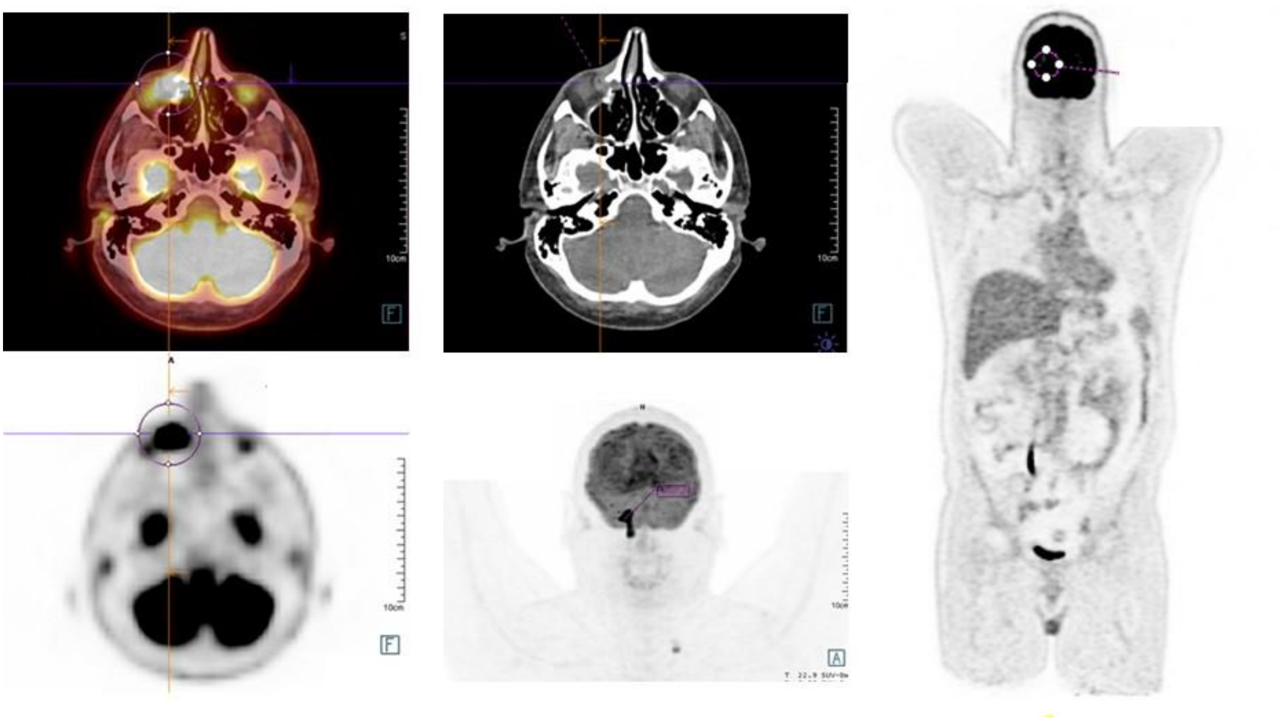

FIGURE S1. Clinical history of the patient. Post-colectomy 18F-FDG-TEP-CT-scan revealing a hematological malignancy (OMS 2008 grade 1-2 follicular lymphoma), diagnosed 3 years before being referred to our department, and involving the lachrymal gland and the bowel.

TABLE S1. Clinical and biological parameters

BCGosis: disseminated BCG disease; CRP: C reactive protein; GFR (MDRD): glomerular filtration rate using the modification of diet in renal disease equation; G-CSF*:* granulocyte-colony

			2017		2018										2019						2020	2021
			**06-13**	**12-01**	**02-01**	**07-23**	**08-02**	**09-12**	**09-27**	**10-10**	**10-26**	**11-02**	**11-07**	**12-15**	**01-11**	**02-22**	**03-29**	**06-07**	**08-08**	**11-22**	**03-10**	**25-01**
**Leucocytes**	4000-10500		5100	3950	4010	3980	3160	1980		2210	3250	2070	2350	5760	6490	5620	3500	3170	3190	2990	3680	4540
**Neutrophils**	1800-7700					3060		1580		1640	2480	1570	1970	4900	5170	4740	2430	2310	2410	2150	2510	3370
**Monocytes**	100-1000							160		170	360	260	210	430	750	410	450	470	430	390	500	360
**Lymphocytes**	1600-2400					530	320	240		190	230	190	200	510	510	420	550	360	362	466	671	618
**CD3+**	1100-1700	/mm^3						197		148	228		174	269	456	366	489	317	316	384	513	419
**CD4+**	700-1100							119		83	127		102	162	283	222	293	186	195	231	296	206
**CD8+**	500-900							49		30	42		38	60	81	72	77	55	55	72	99	76
**CD19+**	200-400							0		0			0	0	0	0	0	0	3	31	71	127
**NK cells**	200-400							5		6			6	16	42	45	57	37	26	25	49	76
**IgG**	7.20-14.70				8.52			7.67				5.5		7.21	8.27	7.93	8.93	8.74	8.96	8.24		8.53
**IgA**	1.10-3.60	g/L						1.06				0.91		1.04	1.07	1.01	1.01	0.81	0.82	0.96		0.86
**IgM**	0.48-3.10							0				0		0	0	0	0	0	0	0.19		0.17
**C reactive protein**	<4	mg/L	38	35	26	31	24	25	58	40	24	11	4.1	0	6	10		7	6.9	19.6	8.9	11.2
**GFR (MDRD)***	>90	ml/min	86	67	53	32	32	26	20	39	46	49	48	53	48	49	51	49	53	53	64	60
**Creatininemia**	64-104	µmol/L				208	206	242	227	172	152	144	146	153	145	143	139	144	133	133	115	120.7
**Proteinuria**		g/L						0.86	0.90	0.82										0.49		
**BCGosis**									**YES**	**YES**	**YES**	**YES**	**YES**	**YES**	**YES**	**YES**	**YES**	NO				
**Pneumocystosis (PCR+)**								**YES**	NO													
**Imukin (recombinant interferon-γ1b)**												**YES**	**YES**	**YES**	**YES**	**YES**	**YES**	**YES**	**YES**	**YES**	**YES**	NO
**G-CSF therapy**											**YES**	**YES**	**YES**	**YES**	**YES**	**YES**	NO					
**IVIg**													NO	**YES**	NO							
**Antibiotherapy of tuberculosis**										**YES**	**YES**	**YES**	**YES**	**YES**	**YES**	**YES**	**YES**	NO				

stimulating factor; IgIV: Intravenous immunoglobulin

TABLE S2. Inborn errors of immunity genes prioritized in WES analysis

ACP5	CD20 (MS4A1)	DNAJC21	IKZF1	MASP2	PRF1	SLC35C1	TNFSF6 (FASL)
ACTB	CD21 (CR2)	DOCK2	IKZF2	MCM4	PRKCD	SLC37A4	TPP1
ADA	CD247	DOCK8	IKZF3	MEFV	PRKDC	SMAD9	TPP2
ADAM17	CD27	EFL1	IKZF4	MKL1	PSEN	SMAD9L	TRAC (TCRa)
ADAR1	CD3D	EIF2AK3	IKZF5	MOGS (GSF1)	PSENEN	SMARCAL1	TRAF3
AICDA	CD3E	ELANE	IL10	MSH6	PSMA3	SMARCD2	TRAF3IP2 (ACT1)
AIRE	CD3G	EPG5	IL10RA	MSN (Moesin)	PSMB4	SP110	TREX1
AK2	CD40	ERCC6L2	IL10RB	MST1 (STK4)	PSMB8	SPINK5	TRNT1
AP1S3	CD40L	EXTL3	IL12B	MTHFD1	PSMB9	SRP54	TTC37
AP3B1	CD45	FAAP24	IL12RB1	MVK	PSMG2	SRY	TTC7A
AP3D1	CD46	FADD	IL17F	MYD88	PSTP1P1 (C2BP1)	STAT1	TYK2
ARHGAP10	CD55	FAM105B (OTULIN)	IL17RA	MYSM1	PSTPIP1	STAT2	UNC13D
ARHGEF1	CD59	FASLG	IL17RB	NBEAL2	PTEN	STAT3	UNC93B1
ARHGEF28	CD70 (TNFSF7)	FAT4	IL17RG	NBS1	PYCARD	STAT5B	UNG
ARHGEF4	CD79a	FCGR3A (CD16)	IL1RN	NCF1	RAB27A	STIM1	USB1
ARPC1B	CD79b	FCN3	IL21	NCF2	RAC2	STN1	USP18
ATM	CD81	FERMT3	IL21R	NCF4	RAG1	STX11	VPS13B
ATP6AP1	CD8A	FEZF2	IL2RA (CD25)	NCKAP1L	RAG2	STXBP2	VPS45
BACH2	CEBPE	FHL1 (COL1A2)	IL2RG	NCSTN	RASGRP1	TAP1	WAS
BCL10	CECR1 (ADA2)	FOXN1	IL36	NEMO (IKBKG)	RBCK1	TAP2	WDR1
BCL11B	CFB	FOXP3	IL36RN	NFAT5	RELA	TAPBP	WIPF1
BLM (RECQL3)	CFD	FPR1	IL7R	NFKB1	RELB	TAZ	XIAP
BLNK	CFH	G6PC3 (SNC4)	INO80	NFKB2	RFX5	TBK1	ZAP70
BTK	CFHR1-5	G6PD	IRAK1	NHEJ1	RFXANK	TBX1	ZNF341
C1QA	CFI	G6PT1	IRAK4	NHP2	RFXAP	TCF3	
C1QB	CFP	GATA1	IRF2BP2	NIK (MAP3K14)	RHOH	TCIRG1	
C1QC	CHD7	GATA2	IRF3	NLRC4	RIPK1	TCN2	
C1R	CIITA	GFI1	IRF7	NLRP1	RLTPR (CARMIL2)	TERC	
C1S	CLEC7A (Dectin-1)	GINS1	IRF8	NLRP12	RMRP	TERT	
C2	CLPB	GINS1	ISG15	NLRP3	RNASEH2A	TFRC	

C3	COPA	HAX1	ITCH	NOD2 (CARD15)	RNASEH2B	THBD	
C4A	CORO1A	HMOX	ITGB2	NOP10	RNASEH2C	THPO	
C4B	CSF2RA	HOIL1 (RPCK1)	ITK	NSMCE3	RNF168	TICAM1	
C5	CSF2RB	HOIP (RNF31)	JAGN1	ORAI-I (ORAI1)	RNU4ARAC	TINF2	
C6	CSF3R	HYOU1	JAK1	OTULIN	RORc	TIRAP	
C7	CTC1	ICF1 (DNMT3B)	JAK2	OX40 (TNFRSF4)	RPSA	TLR3	
C8A	CTLA4	ICF2 (ZBTB24)	JAK3	PARN	RTEL1	TLR7	
C8B	CTPS1	ICF3 (CDCA7)	KDM6A	PEPD	RUNX1	TLR9	
C8G	CTSC	ICF4 (HELLS)	KMT2D	PGM3	SAMHD1	TMC6 (EVER1)	
C9	CXCR2	ICOS	LAMTOR2	PIK3CD	SBDS	TMC8 (EVER2)	
CARD11	CXCR4	IFIH1 (MDA5)	LAT	PIK3R1	SCL46A1	TMEM173 (Sting)	
CARD14	CXCR5	IFNAR2	LCK	PLCG2	SLC39A7	TNFAIP3 (A20)	
CARD9	CYBA	IFNGR1	LIG1	PMS2	SEC61A1	TNFRSF13B (TACI)	
CASP10	CYBB	IFNGR2	LIG4	PNP	SEMA3E	TNFRSF13C (BAFFR)	
CASP8	DCLRE1B	IGHM	LPIN2	POLA1	SERPING1	TNFRSF1A	
CBL	DCLRE1C (Artemis)	IGKC	LRBA	POLE	SH2D1A (XLP1)	TNFRSF6 (FAS)	
CCBE1	DDX58	IGLL1	LYST	POLE2	SH3BP2	TNFSF12 (TWEAK)	
CD19	DKC1	IKBA (NFKBIA)	MAGT1	POMP	SLC29A3		

TABLE S3. Literature review of healthcare-associated disseminated BCG infection in cancer patients without prior BCG instillation

Reference	*Age/Gender*	*Clinical features*	*Chemotherapy regiment or immunosuppressive agent*	*Microbiological identification and histological features*	*Type of BCG contamination*
Meije et al. Clinical infectious diseases 2017 (2)	77y, male	- Colon cancer<br>- Delay between chemotherapy and infection: 8 years<br>- Lung infection (nodules)	ND	Positive PCR and culture in bronchio-alveolar lavage and catheter	Cather-related BCG infection (Onco-Tice strain)
Meije et al. Clinical infectious diseases 2017 (2)	47y, male	- Testicular cancer<br>- Delay between chemotherapy and infection: 9 months<br>- Lung infection (nodules)	ND	Positive PCR and culture in bronchio-alveolar lavage and catheter<br>Non necrotizing granulomas on liver biopsy	Cather-related BCG infection (Onco-Tice strain)
Meije et al. Clinical infectious diseases 2017 (2)	71y, female	- Ovarian cancer<br>- Delay between chemotherapy and infection: 8 years<br>- Lung infection (nodules)	ND	Positive PCR and culture in bronchio-alveolar lavage<br>Non-necrotizing granulomas open air lung biopsy	Cather-related BCG infection (Onco-Tice strain)
Meije et al. Clinical infectious diseases 2017 (2)	69y, female	- Breast cancer<br>- Delay between chemotherapy and infection: 8 years<br>- Lung infection (nodules and infiltrates) and liver nodules	ND	Positive bacilloscopy on transbronchial biopsy and on catheter exudate<br>Positive PCR and culture in bronchio-alveolar lavage and catheter<br>Non-necrotizing granulomatous interstitial pneumonitis on transbronchial biopsy	Cather-related BCG infection (Onco-Tice strain)
Meije et al. Clinical infectious diseases 2017 (2)	80y, male	- Colon cancer<br>- Delay between chemotherapy and infection: 2 years<br>- Lung infection (condensation)	ND	Positive culture in sputum and bronchio-alveolar lavage<br>Non-necrotizing granulomatous interstitial pneumonitis on transbronchial biopsy	Cather-related BCG infection (Onco-Tice strain)
Meije et al. Clinical infectious diseases 2017 (2)	73y, female	- Breast cancer<br>- Delay between chemotherapy and infection: 6 years<br>- Lung infiltrates	ND	Positive culture in bronchial aspirates and lavage	Cather-related BCG infection (Onco-Tice strain)

Meije et al. Clinical infectious diseases 2017 (2)	68y, female	- Breast cancer<br>- Delay between chemotherapy and infection: 4 years<br>- Lung micronodules	ND	Positive PCR and culture in bronchoalveolar aspirate and positive culture in culture<br>Necrotizing granulomatous pneumonitis on transbronchial biopsy	Cather-related BCG infection (Onco-Tice strain)
Meije et al. Clinical infectious diseases 2017 (2)	56y, male	- Colon cancer<br>- Delay between chemotherapy and infection: 10 months<br>- Lung infiltrates	ND	Positive culture in bronchoalveolar aspirate<br>Necrotizing granulomatous pneumonitis on transbronchial biopsy	Cather-related BCG infection (Onco-Tice strain)
Meije et al. Clinical infectious diseases 2017 (2)	62y, female	- Breast cancer<br>- Delay between chemotherapy and infection: 1 year<br>- Lung micronodules	ND	Positive culture in broncho-alveolar aspirate and transbronchial biopsy<br>Necrotizing granulomas on transbronchial biopsy	Cather-related BCG infection (ImmunoCyst® strain)
Coppes MJ et al. Clin Infect Dis 1992 (3)	6y, female	- Acute lymphoblastic leukemia 1 year before the first symptoms<br>- BCG meningitidis	Chemotherapy (Berlin-Franckfurt-Munster 1981 protocol)	Positive identification of acid-tast bacilli on brain biopsy<br>Positivity of PCR in brain biopsy specimen	Not found
Stone MM et al. New England Journal of Medicine 1995 (4)	3y, female	- Acute lymphoblastic leukemia 10 months before<br>- BCG meningitidis<br>- Cutaneous lesion of the back	Chemotherapy (vincristine, prednisone, mercaptopurine, methotrexate as maintenance therapy at the time of BCGosis diagnosis)	Positive culture of cutaneous abscess<br>Positive culture of the cerebrospinal fluid	Not found
Stone MM et al. New England Journal of Medicine 1995 (4)	5y, male	- Acute lymphoblastic leukemia<br>- BCG meningitidis	Chemotherapy (vincristine, prednisone, mercaptopurine, methotrexate as maintenance therapy at the time of BCGosis diagnosis)	Positive culture of cerebrospinal fluid	Not found
Waecker NJ et al. Clin Infect Dis 2000 (5)	2.5y, male	- Down syndrome and acute megakaryocytic leukemia in remission	Chemotherapy	Positive culture of lung biopsy	Not found
Waecker NJ et al. Clin Infect Dis 2000 (5)	13y, female	- Acute lymphocytic leukemia<br>- Remission for 32 weeks since the BCGosis	Chemotherapy	Skin abscess positive to BCG in culture and positivity of direct exam of the	Not found

				biopsy, sputum and bronchoalveolar lavage.	
Waecker NJ et al. Clin Infect Dis 2000 (5)	6y, male	- Acute lymphoblastic leukemia under maintenance therapy (mercaptopurine and methotrexate)	Chemotherapy	Epidural abscess with positive direct examination and positivity of culture to *M. bovis*	Not found
Vos MC et al. J Infect Dis 2003 (6)	11y, female	- Acute lymphoblastic leukemia under chemotherapy and intrathecal instillation<br>- Meningitidis due to BCGosis	Chemotherapy	Positive culture of the cerebrospinal fluid with BCG	Nosocomial (cross contamination with onco-tice during the preparation of the cytotoxic agents used as chemotherapy)
Vos MC et al. J Infect Dis 2003 (6)	13y, female	- Hodgkin disease<br>- Disseminated BCGosis	Chemotherapy	Granuloma in lung biopsy and positive culture of BCG strain in lung biopsy	Nosocomial (cross contamination with onco-tice during the preparation of the cytotoxic agents used as chemotherapy)
Vos MC et al. J Infect Dis 2003 (6)	39y, female	- Disseminated BCGosis	Chemotherapy	ND	Nosocomial (cross contamination with onco-tice during the preparation of the cytotoxic agents used as chemotherapy)
Vos MC et al. J Infect Dis 2003 (6)	30y, male	- AIDS, Burkitt lymphoma<br>- Meningitidis due to BCGosis and disseminated BCGosis	Chemotherapy	Positive culture in the cerebrospinal fluid	Nosocomial (cross contamination with onco-tice during the preparation of the cytotoxic agents used as chemotherapy)
Vos MC et al. J Infect Dis 2003 (6)	4y, female	- Pre-B cell acute lymphatic leukemia	Chemotherapy	Positive culture in bronchoalveolar lavage, granuloma in the bone marrow and positive direct analysis of the bone (osteomyelitis)	Nosocomial (cross contamination with onco-tice during the preparation of the cytotoxic agents used as chemotherapy)

BCG: Bacillus Calmette and Guerin; BCGosis: disseminated BCG disease; ND: no data; AIDS: acquired immunodeficiency syndrome
